# Organelle Simple Sequence Repeat Markers Help to Distinguish Carpelloid Stamen and Normal Cytoplasmic Male Sterile Sources in Broccoli

**DOI:** 10.1371/journal.pone.0138750

**Published:** 2015-09-25

**Authors:** Jinshuai Shu, Yumei Liu, Zhansheng Li, Lili Zhang, Zhiyuan Fang, Limei Yang, Mu Zhuang, Yangyong Zhang, Honghao Lv

**Affiliations:** Institute of Vegetables and Flowers, Chinese Academy of Agricultural Sciences, Key Laboratory of Biology and Genetic Improvement of Horticultural Crops, Ministry of Agriculture, 12 Zhongguancun Nandajie Street, Beijing, 100081, China; Kansas State University, UNITED STATES

## Abstract

We previously discovered carpelloid stamens when breeding cytoplasmic male sterile lines in broccoli (*Brassica oleracea* var. *italica*). In this study, hybrids and multiple backcrosses were produced from different cytoplasmic male sterile carpelloid stamen sources and maintainer lines. Carpelloid stamens caused dysplasia of the flower structure and led to hooked or coiled siliques with poor seed setting, which were inherited in a maternal fashion. Using four distinct carpelloid stamens and twelve distinct normal stamens from cytoplasmic male sterile sources and one maintainer, we used 21 mitochondrial simple sequence repeat (mtSSR) primers and 32 chloroplast SSR primers to identify a mitochondrial marker, mtSSR2, that can differentiate between the cytoplasm of carpelloid and normal stamens. Thereafter, mtSSR2 was used to identify another 34 broccoli accessions, with an accuracy rate of 100%. Analysis of the polymorphic sequences revealed that the mtSSR2 open reading frame of carpelloid stamen sterile sources had a deletion of 51 bases (encoding 18 amino acids) compared with normal stamen materials. The open reading frame is located in the coding region of *orf125* and *orf108* of the mitochondrial genomes in *Brassica* crops and had the highest similarity with *Raphanus sativus* and *Brassica carinata*. The current study has not only identified a useful molecular marker to detect the cytoplasm of carpelloid stamens during broccoli breeding, but it also provides evidence that the mitochondrial genome is maternally inherited and provides a basis for studying the effect of the cytoplasm on flower organ development in plants.

## Introduction

Broccoli (*Brassica oleracea* L. var *italica*), sometimes referred to as Calabrese, is the most important commercial form of *Brassica* [1]. Driven by its reported abundance of nutrients and health benefits, broccoli has been well received by consumers. Its production and consumption has risen sharply in recent years [2–10], especially in the United Kingdom. Broccoli has an important commercial position, with a planting area of more than 7,000 hectares and a production value of more than £50 million (approximately US$77 million) each year [1, 11].

Broccoli displays obvious heterosis: using a cytoplasmic male sterile (CMS) line and an inbred line to create hybrids can reduce the cost of producing hybrids and improve their purity. However, hybrid production is difficult and produces low yields; therefore, CMS sources are crucial in improving the yield of hybrids. The CMS sources of broccoli have mostly been transferred from cabbage, radish, and other related cruciferous species through backcrossing. However, the floral organs often show extremely complex morphological variations because of the lack of coordination between the nucleus and cytoplasm during the transfer process [12–13]. These variations are different among varying genetic backgrounds in broccoli [14], thereby increasing the difficulty of transferring CMS materials. We previously found that CMS lines of broccoli obtained through some male sterile sources displayed carpelloid stamens. When we used these lines to produce hybrids, the pods were abnormal and seed yields were very low. These consequences dramatically limit the use value of these lines.

Carpelloid stamen phenomena have been studied in *Arabidopsis thaliana* and are regulated by class B genes of the classic ABC model. If the MADS-box transcription factors *APETALA3* (*Ap3*) or *PISTILLATA* (*PI*), which are class B genes involved in conferring identities of the stamen and petal, are mutated or deleted, homeotic conversions of stamens to carpels and petals to sepals can occur [15–17]. Furthermore, the carpelloid stamen phenomenon has been reported in *B*. *juncea* var. *tumida* [18], *B*. *rapa* subsp. *chinensis* [19–20], *B*. *napus* [21–23], *B*. *juncea* [24], and *Daucus carota sativus* [25]. Thus far, carpelloid stamens caused by allo-cytoplasmic inheritance have not been documented in CMS lines of broccoli.

Mitochondrial and chloroplast genes are mainly inherited in a maternal pattern, with a slow rate of mutation; therefore, mitochondrial and chloroplast DNA have been widely used in evolutionary and phylogenetic studies [26–31]. The mitochondrial genome has a complicated multipartite structure [32], whereas the structure of the chloroplast genome is more conserved [28, 33–35]. To date, the entire mitochondrial genomes of *Arabidopsis thaliana*, *B*. *napus* (Nap and Pol), *B*. *rapa* (Cam), *B*. *oleracea*, *B*. *juncea*, and *B*. *carinata* have been determined [36–39]. The complete chloroplast genomes of *Arabidopsis thaliana* and *B*. *napus* have also been determined [40–41]. Owing to the large number of mitochondrial and chloroplastic sequences available, more and more markers have been developed to analyze genetic diversity [42–48] and variation of cytoplasmic [49–52] and CMS types [53–55]. However, as far as we know, mitochondrial and chloroplastic markers have not been used to distinguish between carpelloid and normal stamens in broccoli. The objectives of this study were: (1) to understand the inheritance pattern and morphological characteristics of carpelloid stamens; (2) to develop chloroplast and mitochondrial simple sequence repeat (SSR) markers that can distinguish between CMS sources of carpelloid and non-carpelloid stamens in broccoli; (3) to confirm the sequence features of the polymorphic bands by cloning and sequencing; and (4) to analyze the similarity of the genes related to carpelloid stamens.

## Materials and Methods

### Plant material and DNA extraction

The plant materials used in this study are detailed in Table 1. In total, 51 broccoli accessions, including 19 CMS lines and 23 hybrids were studied. Nine high-generation maintainers were included as references, and the backcross generations of CMS lines were continuously backcrossed to 2014.

**Table 1:** List of 51 broccoli accessions used in this study and their stamen types, identified by a PCR assay.

Code	Line name	Type	Backcross generations	Origin	Stamens state
B1	93219	Inbred line	-	Institute of Vegetables and Flowers, Chinese Academy of Agricultural Sciences (Beijing, China)	Normal
B2	CMS0412×93219	Cytoplasmic male sterile line	8	Institute of Vegetables and Flowers, Chinese Academy of Agricultural Sciences (Beijing, China)	Normal
B3	CMS0413×93219	Cytoplasmic male sterile line	8	Institute of Vegetables and Flowers, Chinese Academy of Agricultural Sciences (Beijing, China)	Normal
B4	CMS05738×93219	Cytoplasmic male sterile line	7	Institute of Vegetables and Flowers, Chinese Academy of Agricultural Sciences (Beijing, China)	Normal
B5	CMS04S132×93219	Cytoplasmic male sterile line	8	Institute of Vegetables and Flowers, Chinese Academy of Agricultural Sciences (Beijing, China)	Carpellody
B6	CMS10Q688×93219	Cytoplasmic male sterile line	2	Institute of Vegetables and Flowers, Chinese Academy of Agricultural Sciences (Beijing, China)	Carpellody
B7	LvFu	Hybrid	-	Variety introduction (Wong Ching Ho Co., Ltd., Hong Kong, China)	Normal
B8	BT-2006	Hybrid	-	Variety introduction (Beijing Honor Seeds Co., Ltd., Beijing, China)	Normal
B9	BT-2007	Hybrid	-	Variety introduction (Beijing Honor Seeds Co., Ltd., Beijing, China)	Normal
B10	L×2J	Hybrid	-	Variety introduction (Tianjin Kernel Vegetable Research Institute, Tianjin, China)	Normal
B11	L×FJ	Hybrid	-	Variety introduction (Tianjin Kernel Vegetable Research Institute, Tianjin, China)	Normal
B12	JingYou	Hybrid	-	Variety introduction (Wong Ching Ho Co., Ltd., Hong Kong, China)	Normal
B13	NaiHanYouXiu	Hybrid	-	Variety introduction (Sakata Seed Corporation, Japan)	Normal
B14	Tie mountain	Hybrid	-	Variety introduction (Seminis Seeds Beijing Co., Ltd., Beijing, China)	Normal
B15	HeHuan007	Hybrid	-	Variety introduction (Ho-Huan Agricultural Product Co., Ltd., Taiwan, China)	Carpellody
B16	SaLi’Ao 55	Hybrid	-	Variety introduction (Syngenta China Company, Beijing, China)	Normal
B17	NanXiu366	Hybrid	-	Variety introduction (Seminis Seeds Beijing Co., Ltd., Beijing, China)	Carpellody
B18	8554	Inbred line	-	Institute of Vegetables and Flowers, Chinese Academy of Agricultural Sciences (Beijing, China)	Normal
B19	90196	Inbred line	-	Institute of Vegetables and Flowers, Chinese Academy of Agricultural Sciences (Beijing, China)	Normal
B20	93213	Inbred line	-	Institute of Vegetables and Flowers, Chinese Academy of Agricultural Sciences (Beijing, China)	Normal
B21	94177	Inbred line	-	Institute of Vegetables and Flowers, Chinese Academy of Agricultural Sciences (Beijing, China)	Normal
B22	YN23	Inbred line	-	Institute of Vegetables and Flowers, Chinese Academy of Agricultural Sciences (Beijing, China)	Normal
B23	YN36	Inbred line	-	Institute of Vegetables and Flowers, Chinese Academy of Agricultural Sciences (Beijing, China)	Normal
B24	05726	Inbred line	-	Institute of Vegetables and Flowers, Chinese Academy of Agricultural Sciences (Beijing, China)	Normal
B25	05732	Inbred line	-	Institute of Vegetables and Flowers, Chinese Academy of Agricultural Sciences (Beijing, China)	Normal
B26	CMS0412×93219	Cytoplasmic male sterile line	BC_9_	Institute of Vegetables and Flowers, Chinese Academy of Agricultural Sciences (Beijing, China)	Normal
B27	CMS0413×93219	Cytoplasmic male sterile line	BC_9_	Institute of Vegetables and Flowers, Chinese Academy of Agricultural Sciences (Beijing, China)	Normal
B28	CMS05738×93219	Cytoplasmic male sterile line	BC_8_	Institute of Vegetables and Flowers, Chinese Academy of Agricultural Sciences (Beijing, China)	Normal
B29	CMS04S132×93219	Cytoplasmic male sterile line	BC_6_	Institute of Vegetables and Flowers, Chinese Academy of Agricultural Sciences (Beijing, China)	Carpellody
B30	CMS04S132×93219	Cytoplasmic male sterile line	BC_7_	Institute of Vegetables and Flowers, Chinese Academy of Agricultural Sciences (Beijing, China)	Carpellody
B31	CMS04S132×93219	Cytoplasmic male sterile line	BC_9_	Institute of Vegetables and Flowers, Chinese Academy of Agricultural Sciences (Beijing, China)	Carpellody
B32	CMS04S132×YN36	Cytoplasmic male sterile line	BC_6_	Institute of Vegetables and Flowers, Chinese Academy of Agricultural Sciences (Beijing, China)	Carpellody
B33	CMS10QB688×93219	Cytoplasmic male sterile line	BC_3_	Institute of Vegetables and Flowers, Chinese Academy of Agricultural Sciences (Beijing, China)	Carpellody
B34	CMS10QB688×90196	Cytoplasmic male sterile line	BC_2_	Institute of Vegetables and Flowers, Chinese Academy of Agricultural Sciences (Beijing, China)	Carpellody
B35	CMS10QB688×90196	Cytoplasmic male sterile line	BC_3_	Institute of Vegetables and Flowers, Chinese Academy of Agricultural Sciences (Beijing, China)	Carpellody
B36	CMS10QB688×93213	Cytoplasmic male sterile line	BC_2_	Institute of Vegetables and Flowers, Chinese Academy of Agricultural Sciences (Beijing, China)	Carpellody
B37	CMS10QB688×93213	Cytoplasmic male sterile line	BC_3_	Institute of Vegetables and Flowers, Chinese Academy of Agricultural Sciences (Beijing, China)	Carpellody
B38	CMS10QB688×94177	Cytoplasmic male sterile line	BC_2_	Institute of Vegetables and Flowers, Chinese Academy of Agricultural Sciences (Beijing, China)	Carpellody
B39	CMS10QB688×94177	Cytoplasmic male sterile line	BC_3_	Institute of Vegetables and Flowers, Chinese Academy of Agricultural Sciences (Beijing, China)	Carpellody
B40	(CMS04S132×05732) ×YN23	Hybrid	-	Institute of Vegetables and Flowers, Chinese Academy of Agricultural Sciences (Beijing, China)	Carpellody
B41	(CMS04S132×YN23) _6_ ×93219	hybrid	-	Institute of Vegetables and Flowers, Chinese Academy of Agricultural Sciences (Beijing, China)	Carpellody
B42	(CMS04S132×YN36) _6_ ×93219	hybrid	-	Institute of Vegetables and Flowers, Chinese Academy of Agricultural Sciences (Beijing, China)	Carpellody
B43	(CMS04S132×93219 _5_×YN26_3_)×05732	Hybrid	-	Institute of Vegetables and Flowers, Chinese Academy of Agricultural Sciences (Beijing, China)	Carpellody
B44	(CMS04S132×93219) _5_×YN36×05726	Hybrid	-	Institute of Vegetables and Flowers, Chinese Academy of Agricultural Sciences (Beijing, China)	Carpellody
B45	(CMS04S12×93219) _4_ ×YN36	Hybrid	-	Institute of Vegetables and Flowers, Chinese Academy of Agricultural Sciences (Beijing, China)	Carpellody
B46	HeHuan007×8554	Hybrid	-	Institute of Vegetables and Flowers, Chinese Academy of Agricultural Sciences (Beijing, China)	Carpellody
B47	HeHuan007×93213	Hybrid	-	Institute of Vegetables and Flowers, Chinese Academy of Agricultural Sciences (Beijing, China)	Carpellody
B48	HeHuan007×93219	Hybrid	-	Institute of Vegetables and Flowers, Chinese Academy of Agricultural Sciences (Beijing, China)	Carpellody
B49	NanXiu366×8554	Hybrid	-	Institute of Vegetables and Flowers, Chinese Academy of Agricultural Sciences (Beijing, China)	Carpellody
B50	NanXiu366×93213	Hybrid	-	Institute of Vegetables and Flowers, Chinese Academy of Agricultural Sciences (Beijing, China)	Carpellody
B51	NanXiu366×93219	Hybrid	-	Institute of Vegetables and Flowers, Chinese Academy of Agricultural Sciences (Beijing, China)	Carpellody

Note

‘-’ means no backcross generations.

These accessions were grown in experimental greenhouses under standard field conditions at the Institute of Vegetable and Flowers, Chinese Academy of Agricultural Sciences, Changping, Beijing, China, from 2011 to 2014. When the diameter of the main bouquet on each plant was 8–10 cm, we pruned the bouquet, leaving three lateral balls, and then left the plant to develop branches naturally. Plantlets at the eight to ten leaves stage were randomly chosen from each accession for total genomic DNA isolation, using the modified hexadecyltrimethylammonium bromide method [56].

### Flowering and fruiting characteristics observation

When the plants produced flowers, pollination was performed by hand or naturally (free pollination using bees in hives placed along the greenhouse, at positions corresponding to 1/8, 3/8, 5/8, and 7/8 of its length, from early flowering to the end of flowering) in 2011–2014. Flower morphologies, the pod shapes of each material, and the grain number per pod of maintainer 93219 (B1; Table 1) and different generations of CMS line CMS0412×93219, CMS0413×93219, CMS05738×93219, and CMS04S132×93219 (B2–B5, B26–B31; Table 1) were observed.

### PCR and sequence analysis

Twenty-one pairs of primers for *Brassica napus* mitochondrial genome sequences (Gen Bank GI: 112253843) and 32 pairs of primers for *Arabidopsis thaliana* chloroplast sequences (Gen Bank GI: 7525012) developed by Zhang [57] were used to screen for makers that distinguished carpelloid from normal stamens (Table 2). All primers were synthesized by Sangon Biotech Co., Ltd (Shanghai, China).

**Table 2:** PCR primers used in this study.

Name	Forward primer 5′-3′	Reverse primer 5′-3′
ACP1	GAACGACGGGAATTGAACC	GGTGGAATTTGCTACCTTTTT
ACP2	GAAAAATGCAAGCACGGTTT	TACGATCCGTAGTGGGTTGC
ACP4	TACCCGTATTAGGCACTA	TTTGTAAGACCACGACTG
ACP7	TGGAGAAGGTTCTTTTTCAAGC	CGAACCCTCGGTACGATTAA
ACP9	AACCATAATCATAGAAATAGAG	GTCGAACAAAGTAATCGG
ACP10	GTATTAAATCCGAAACTC	ACTTGACATAAAACTTGG
ACP15	CGACCAATCCTTCCTAAT	GAATGTTTGCTACCCTGA
ACP17	TGCACTCTTCATTCTCGTTCC	GCGTTCCTTTCATTTAAGACG
ACP18	AATGAAGAGTGCAGTAGC	CTAGGTTTTAGAAAGGAAA
ACP20	CTCAACCGCCATCATACT	CGAAACTTAACCCTCTTT
ACP25	CCCAAACCAAAGAGTGTA	GCTCGCAAGGCTCATAAC
ACP26	AGAGGACCAAGAAACCAA	AACAGGCCATTCAACAAG
ACP27	GAAGAGCAAACAAGGGAT	GAAGGGTTAGTCAATCAAAT
ACP29	GGCCATAGGCTGGAAAGTCT	GTTTATGCATGGCGAAAAGG
ACP32	TTCATAAGCGAAGAACAA	TCAGAGTAAGCAAAACAT
ACP33	AGGGATAGTAATAAGAATAG	CAGATGTAAGAACGAAAA
ACP35	ATTGGCTTACTTCTTGCG	GGTTTCCGGGATGTTATT
ACP38	GGTTCGTTAGCAGGTTTA	GTTCCCTAGCAACACTTT
ACP39	AGACGGGTGAATAGAGTG	GTTATGCTTTTCGACGAT
ACP40	GCAGAATGAGACGGGATA	AGACACTTTGGGATTGCT
ACP41	GGCTCCACAATGGAATTGAC	GCACATTTCAGCGTCACAAA
ACP42	CTTTCTCGATAAAGTCGGTTGA	GGAAGAAGCCCGTTCAGG
ACP43	GCTGTCGTGGATGAGTGG	AAGTGCTTTCTGGGTCGT
ACP44	ATTGTAGATTCTGGGAGG	ATCGATGCTGTATTCATG
ACP45	TTACATTGCTTTTCTTACAG	CTCGTTGGTTTAGGATTA
ACP46	CTACCATTTCACCACCAA	GGACCCTATTCACCTCTT
ACP47	TGTACTTATGGGAAAGCG	CTGGGTTCTTCTACTTCATT
ACP48	AGGCAAGATGATAGGATA	CAAGTCAAGATGATACGG
ACP49	AATAGCTCGACGCCAGGAT	TTCGGATGTGAAAGTGCC
ACP50	TCCGAGTGAATGGAAAAG	GATGGAATTACAAATGGAGTAG
ACP51	TTCTTATTCACTGGTTTG	GTGGAAATCTTTGTTCTA
ACP58	GCTATCCCAAGTTTCTGC	TGGCTTTGATCCGTTATT
mtSSR1	CTCCCGCTTCCTCACATC	GCTCCAATAAGGCGTTCC
mtSSR2	ACCAAGATTGAGCCAGAT	CGTCCACTACCGAAAGAG
mtSSR3	GGCTGCTTTCTCATACCG	TCCTAAATGCTGCCCTTC
mtSSR4	GATTCTAAGGGTACGGGACA	ACCGACGACAAACAATAACA
mtSSR5	TATTGTTTGTCGTCGGTTAT	GGTAGGCAAGTTGGTAGG
mtSSR7	CGTTAGGGGTATTTAGCA	CTCTATTCCGTTTCCACA
mtSSR8	CCCGAGAAGCACTGTTGA	ACGGAGTGACAAAGGAGC
mtSSR9	CGGTGAAAGAGGGCAAAG	AAACAAATACCAGCTAACG
mtSSR10	GCCATTTCATTTCCTTTG	ATCCTCCTTCGCCTTTCT
mtSSR22	TACTAATCGGTGACTTGCT	AGTCTTTATGGATGTGCC
mtSSR46	TACTTGCTGCACTTCCTG	ACAAATGCCACTTCTTCC
mtSSR48	CCTTCTGGGTTGACTTGA	AGTGGTGCCCTCCTCTAA
mtSSR61	GGCGGTGGACAGAAATGG	AGGGAAGCCCAACGAATG
mtSSR92	GCCGCTTTCATTGTTGTA	TTCGGTTTATTAGCTCTTCC
mtSSR98	GTGCCAGATGCAACAAAG	GAGGCCATAGGGAAAGTC
mtSSR110	CGGGTGCTTGCATCATTT	TCTAGCCATTCCAGGTTT
mtSSR128	AATCCTATCCCATCCGAGTC	AGCCTTTCCTTTCCCACC
mtSSR129	CTTCCCTCAGTTGGTTTG	TGCCCTCTGTCCTTTATT
mtSSR133	GCTGCTCATCACTACCTG	CACTACGCTCACTGAAACTA
mtSSR152	AAGAAAGAAGAGCGACAA	GGGTACGGTACTAAAGGT
mtSSR156	TACTCATCAAATGGCACTC	CAAAGGGAAAGAAGAAAG

PCR amplifications were carried out in a reaction volume of 25 μL, which contained 2 μL (30 ng μL^−1^) genomic DNA, 12.5 μL Dream Taq™ Green PCR Master Mix (Thermo Fisher Scientific Inc., Waltham, MA, USA), and 1 μL of each primer (10 pmol μL^−1^). The following amplification protocol was carried out in an ABI Veriti 96-well PCR thermal cycler (Applied Biosystems, Foster City, CA, USA). Initial denaturation was performed at 94°C for 4 min; followed by 35 cycles of denaturation at 94°C for 30 s, annealing at 55°C for 30 s, and extension at 72°C for 30 s; and a final extension at 72°C for 10 min. PCR products were analyzed on 2.0% (w/v) agarose gels in 0.5×TBE buffer and visualized after staining with Goldview (Solarbio Technology Co., Ltd, Beijing, China). The bands were photographed under ultraviolet light under a Universal Hood II (Bio-Rad, Hercules, CA, USA).

For each single polymorphic locus, the cloned products were sequenced by Bo Maide Biotech Co., Ltd, Beijing, China. Alignment and similarity analyses of the obtained sequences were performed using the Align and BLAST tools, respectively, in the Universal Protein Resource (UniProt) database.

## Results

### Flowering and fruiting characteristics of carpelloid stamen plants

To determine the mechanism of inheritance of the carpelloid stamen phenomenon, we carried out crosses or successively backcrosses between maintainer lines with normal stamens (B1, B18, B19, B20, B21; Table 1) and CMS lines with carpelloid stamens (B6, B15, B17, B29; Table 1) materials in 2011–2014. The results showed that the offspring obtained by crossing or backcrossing all had carpelloid stamens. Therefore, the phenomenon of CMS carpelloid stamens was caused by allo-cytoplasmic inheritance in broccoli; i.e., it was mainly inherited in a maternal pattern and the heritability rate was 100%.

**Fig 1:**
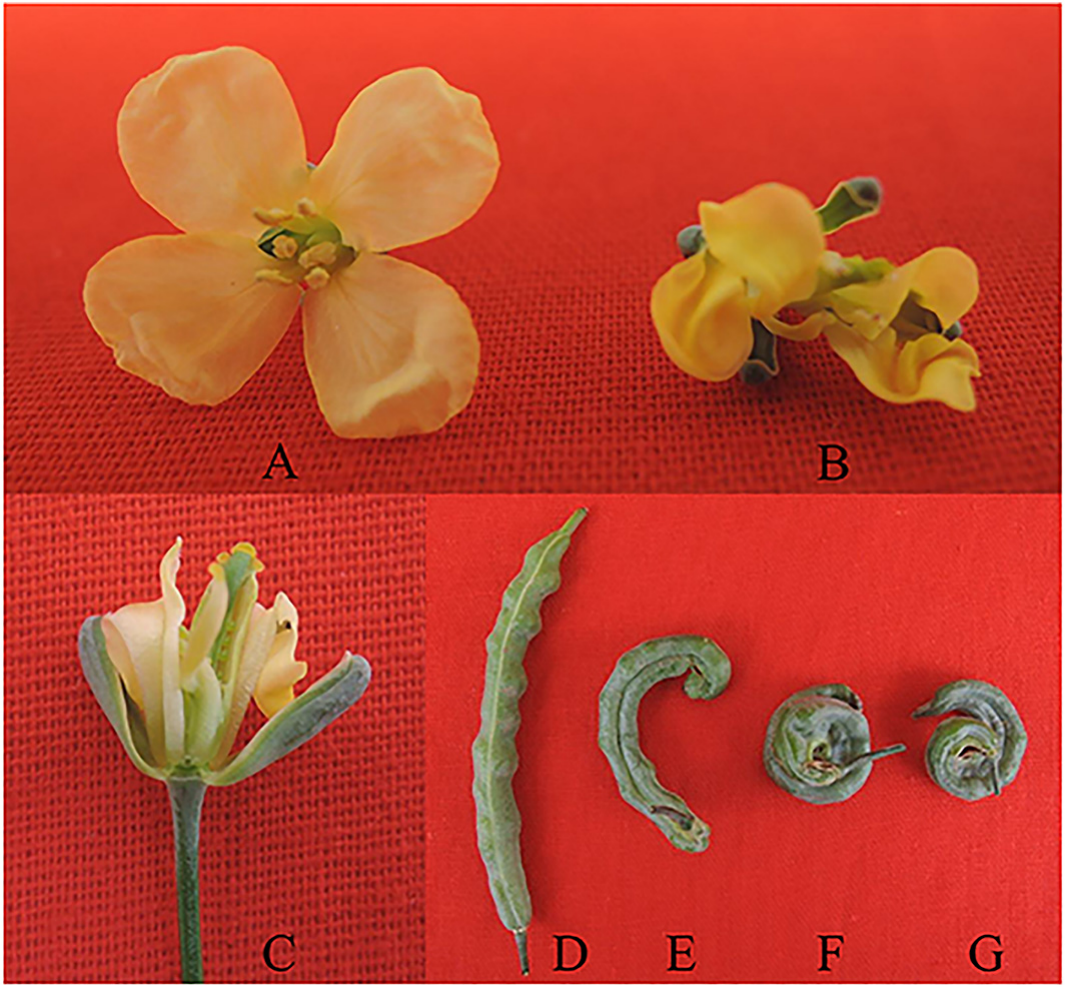
Morphological characteristics of flowers and siliques of maintainer 93219 and CMS04S132×93219. A: flower of maintainer 93219, B and C: flowers of CMS04S132×93219, D: siliques of maintainer 93219, E–G: siliques of CMS04S132×93219.

The growth characteristics of maintainer 93219 (B1; Table 1) and the sixth generation to ninth generation of CMS04S132×93219 (B5, B29, B30, B31; Table 1) plants were observed from 2011 to 2014. The growth and morphological characteristics of the two lines were similar from seed germination to plant bolting, but differences appeared from the big bud stage to before early flowering. In maintainer 93219 plants, the buds were plump with smooth surfaces, and the floral organs developed normally, with six stamens and opened petals (Fig 1A). In addition, the pistils and seed pods were all erect (Fig 1D) with 12.72 ± 0.86 grains per silique. In the different generations of CMS04S132×93219, the buds were soft with slightly wrinkled surfaces, the flowers showed developmental abnormalities, the petals were smaller and deformed, and three to six stamens appeared that strongly varied (Fig 1B and 1C). Interestingly, the stamens mutated into carpelloid structures and pseudo pistils, which were entangled with the pistil; some small green beads, resembling ovules, were observed inside the stamens (Fig 1C). The carpelloid stamens adhered to the pistil, causing the pistil to bend before flowering. The stamens unfolded gradually with flowering; however, most could not completely separate from the pistil. Moreover, the plants could fruit via artificial pollination during the bud and flowering stages. The small green beads in the stamens stopped growing and died away as the siliques developed. The siliques were hooked or coiled (Fig 1E–1G) and were set at a rate of 80.90 ± 3.30% per plant and 6.10 ± 0.53 grains per silique. The number of siliques per plant was much lower than the other CMS lines (B2, B3, and B4; Table 1) that had undergone free pollination.

### Identification of carpelloid stamens of broccoli CMS materials using cpSSR and mtSSR molecular markers

**Fig 2:**
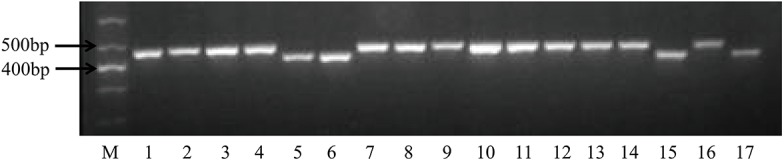
PCR amplification profiles of 17 broccoli accessions. M: marker, 1: maintainer 93219, 2: CMS0412×93219_8_, 3: CMS0413×93219_8_, 4: CMS05738×93219_7_, 5: CMS04S132×93219_8_, 6: CMS10Q688×93219_2_, 7: LvFu, 8: BT-2006, 9: BT-2007, 10: L×2J, 11: L×FJ, 12: JingYou, 13: NaiHanYouXiu, 14: Tie mountain, 15: HeHuan007, 16: SaLi’Ao 55, 17: NanXiu366.

We first used 32 pairs of chloroplastic SSR (cpSSR) primers and 21 pairs of mitochondrial SSR (mtSSR) primers developed by Zhang [57] (Table 2) to amplify the total genomic DNA of five CMS lines (B2–B6; Table 1), maintainer 93219 (B1; Table 1), and 11 unequal hybrids of CMS lines (B7–B17; Table 1), which were collected from seven countries. The results demonstrated that all the cpSSR primers and mtSSR primers could successfully amplify a product. However, only one mtSSR primer (mtSSR2) showed obvious polymorphisms between normal and carpelloid stamen materials (Fig 2). We then used the primers of mtSSR2 to screen the other 34 broccoli accessions (B17–B51; Table 1), including eight inbred lines, 14 CMS lines, and 12 CMS hybrids. The results showed that mtSSR2 could distinguish between the normal and carpelloid stamen materials with 100% accuracy.

### Sequence features of polymorphic bands

**Fig 3:**
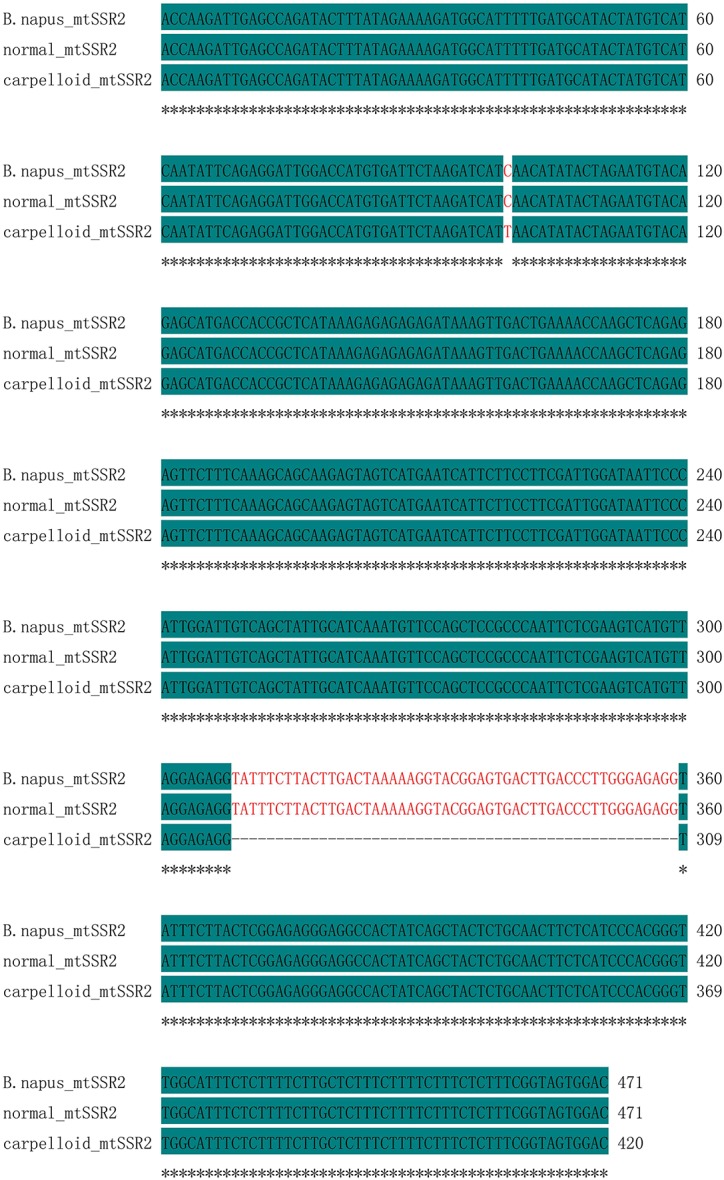
Sequence alignment of mtSSR2 amplicons with the *B*. *napus* mitochondrial genome.

**Fig 4:**
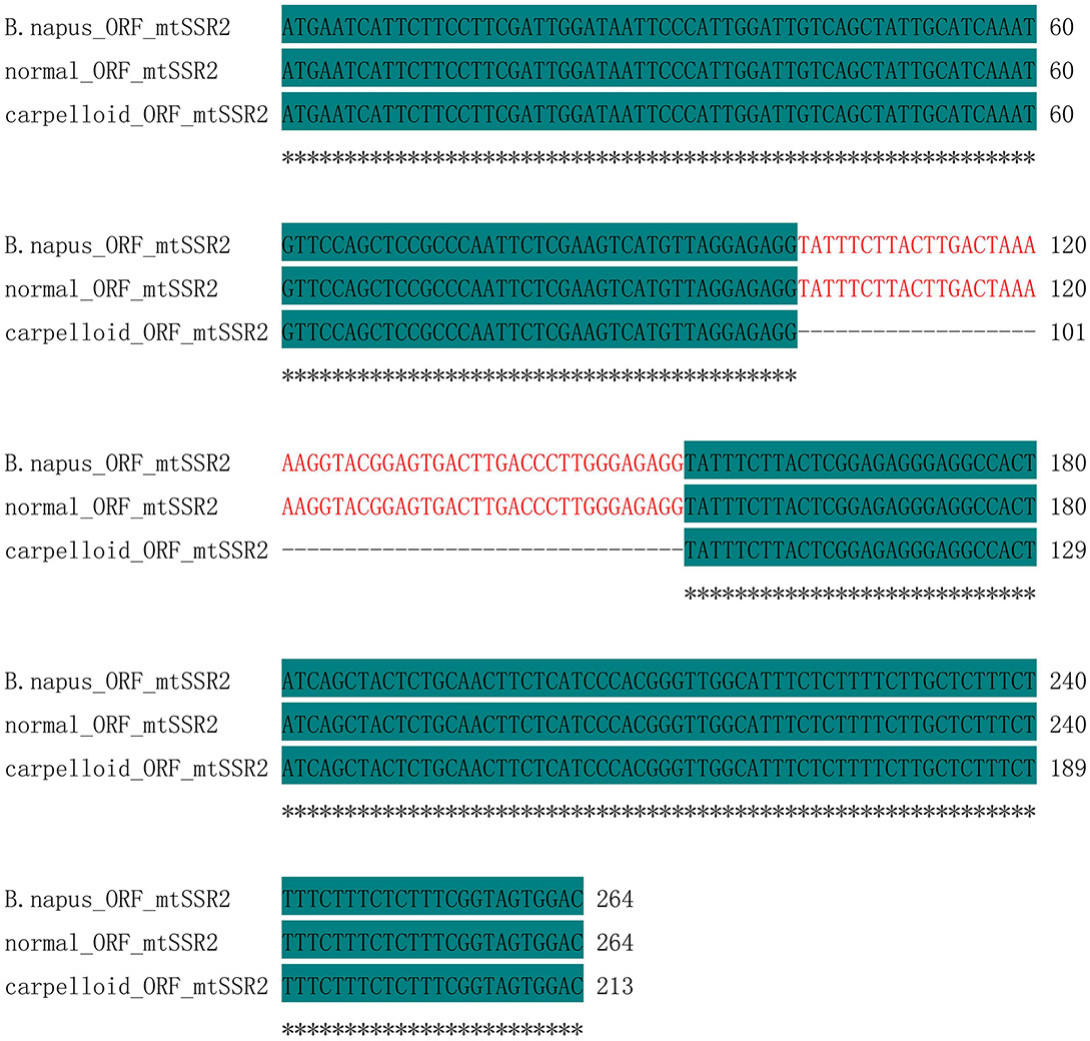
ORF sequence alignment of mtSSR2 amplicons with the *B*. *napus* mitochondrial genome.

**Fig 5:**
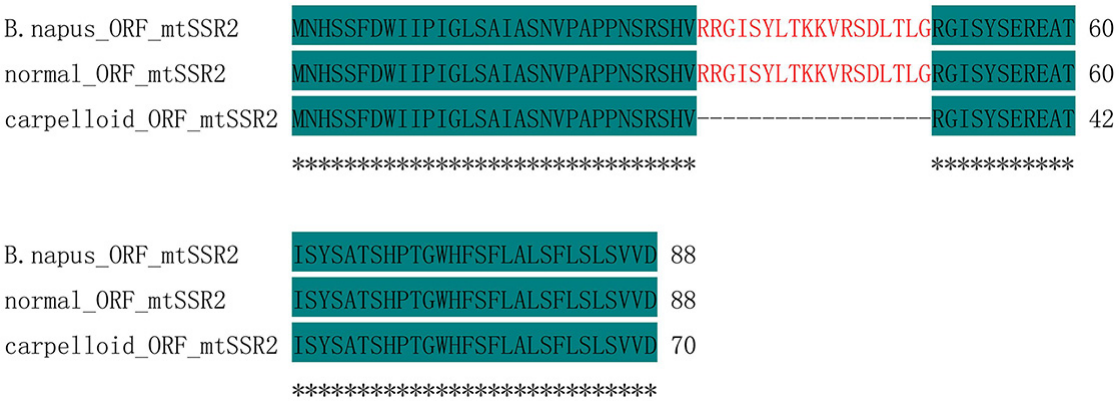
Amino acid sequences of ORFs of mtSSR2 amplicons and the *B*. *napus* mitochondrial genome.

The polymorphic bands obtained after PCR using mtSSR2 were sequenced. The amplicons were identical in all maintainer lines (471 bp), whereas they were 420 bp in all carpelloid stamen materials. Analyses after aligning the polymorphic sequences from normal and carpelloid materials indicated that the similarity of the sequences were 88.96%, with one single nucleotide polymorphism at 100 bp (C/T), and the sequences from carpelloid stamen materials had 51 nucleotides deleted between 309 and 359 bp compared with the normal sequences (Fig 3). Open reading frame (ORF) analysis showed that the deleted nucleotides were located between position 102 and 152 bp in the ORF coding region of the normal sequences (Fig 4), and encoded 18 amino acids (Fig 5). These results indicated that the polymorphism of mtSSR2 could be attributed to the fragment insertion or deletion near the SSR loci.

### Analyzing similarity of genes related to carpelloid stamens

Analysis of the amino acid sequences in the ORF region demonstrated that the polymorphic region amplified with mtSSR2 is located in the *orf125* coding region of *B*. *napus*, *B*. *juncea*, *B*. *rapa* subsp. *oleifera*, *Eruca sativa*, *B*. *oleracea*, *B*. *oleracea* var. *botrytis*, and *B*. *juncea* var. *tumida*. It is also located in the coding region of *orf108c* and *orf108* in *B*. *carinata* and *Raphanus sativus*, respectively. However, the proteins were annotated as being hypothetical and as having unknown functions. Similarity analysis of the amino acid sequences revealed that the protein sequences related to the carpelloid stamen have the highest similarity with *orf108c* and *orf108* in *B*. *carinata* and *Raphanus sativus*, respectively (Table 3).

**Table 3:** Sequence identity between amplified products of mtSSR2 in broccoli CMS normal and carpelloid stamen materials and the corresponding sequences of *B*. *juncea*, *B*. *rapa*, *Eruca sativa*, *B*. *oleracea*, *B*. *oleracea* var. *botrytis*, *B*. *juncea* var. *tumida*, *B*. *napus*, *B*. *carinata*, and *Raphanus sativus* cytoplasmic genomes.

Entry	Organism	Protein name	Sequence identity (normal stamen)	Sequence identity (carpelloid stamen)
G4XYV8	*B*. *juncea*	Orf125	100%	79.5%
G4XYB0	*B*. *rapa* subsp. *oleifera*	Orf125	100%	79.5%
A0A088BGJ2	*Eruca sativa*	Orf125	100%	79.5%
G4XYC4	*B*. *oleracea*	Orf125	100%	79.5%
A0A068BCT7	*B*. *oleracea* var. *botrytis*	Orf125	100%	79.5%
A0A023VX75	*B*. *juncea* var. *tumida*	Orf125	100%	79.5%
Q6YSR6	*B*. *napus*	ORF125	100%	79.5%
G4XYQ4	*B*. *carinata*	Orf108c	80.7%	98.6%
R4I1C8	*Raphanus sativus*	Orf108	80.7%	98.6%

## Discussion

### Carpelloid stamen phenomenon and its inheritance pattern

In higher plants, the process of flowering is complex, involving many interactions between genes or between genes and the environment. The carpelloid stamen phenomenon refers to stamen structures of the flower that are converted to carpels. It not only causes alteration of the flower structure, but also brings about male sterility. This phenomenon is caused by floral homeotic mutations and is controlled by nuclear genes. The B class or C class genes, such as *AP3*, *PI*, *AGL8*, *SHP1*, *SHP2*, and *NAP*c, are involved in the regulation of this phenomenon [15–16, 20, 23, 58–62]. The effect of the plasmon or interactions between cytoplasmic and nuclear genes on the development of floral organs has been reported [14, 21, 25, 63]; however, the molecular mechanisms are not clear. In this study, we confirmed that the carpelloid stamens were caused by allo-cytoplasmic inheritance in CMS lines of broccoli, using hybrid and backcross methods combined with field observation for several years. Stamen carpellody was passed on in a maternal inheritance fashion. Furthermore, the phenotypic features of the carpelloid stamen materials were similar to the carpellody of stamens in *B*. *napus* and *B*. *rapa* obtained from the mutation of nuclear genes: the flowers showed dysplasia, the petals were smaller and deformed, the stamens were seriously deformed, the pistil was deviated, and the stamens were entangled with the pistil [20, 23]. The siliques were erect and the seed setting and combining ability performed well in CMS carpelloid stamen materials of *B*. *napus* and *B*. *rapa*. In contrast, the siliques were hooked or coiled, with few grains per silique observed in this study, which would reduce the utility of CMS lines in hybrid seed production practice in broccoli. Therefore, the occurrence of carpelloid stamens should be avoided in broccoli breeding.

### Maternal inheritance and application of the organelle SSR markers

Chloroplasts and mitochondria are mainly inherited uniparentally in seeded plants [64]. The chloroplast genome is usually maternally inherited in most angiosperm species, including cruciferous crops, such as cabbage, broccoli, and cauliflower [47, 65], although it can also be paternally [66–67] or biparentally inherited [68–69]. Mitochondrial DNA is maternally inherited or passed via paternal transmission [67]. However, mitochondrial inheritance in broccoli has not been documented so far, perhaps because no polymorphisms have been found. In our study, mtSSR2 could be detected in carpelloid stamen materials for four successive backcross generations and there was no sequence variation in the mitochondrial PCR products studied. Our results provide evidence not only that the mitochondrial genome is maternally and stably inherited in broccoli, but also that broccoli mtDNA has advantages for evolutionary studies.

The main advantage of organelle genomes is that no recombination can occur between two alleles, because there is only one allele per cell and per organism [69]. Furthermore, organelle DNA can be easily obtained because it exists in many copies in each cell. Based on organelle genomes’ inheritance patterns and characteristics, organelle SSR markers have been used widely with total genomic DNA. Kaundun and Matsumoto [70] used cpSSR markers to analyze the variation of heterologous nuclear and chloroplast DNA in tea (*Camellia sinensis*). Zhang et al. [47] studied the inheritance of the cabbage chloroplast and assessed subspecies diversity of *B*. *oleracea* using cpSSR primers. Cheng et al. [71] distinguished somatic hybrids in citrus using cpSSR primers. Moreover, cpSSR markers have also been used in neotropical orchids [72] and cruciferous crops [42, 44, 48, 55]. For mitochondrial SSR markers, Wang et al. [55] used mtSSR primers to distinguish allo-cytoplasmic inheritance in cabbage. To the best of our knowledge, the identification of carpelloid and normal stamens using cpSSR or mtSSR in broccoli, as well as other species, has not been reported. In this study, mtSSR2 could accurately distinguish carpelloid from normal stamen materials with the same polymorphic products as reported in cabbage, which can distinguish OguCMSR_1-2_, OguCMSHY, and NigCMS (420 bp) from pol CMS and OguCMSR_3_ (471 bp) [55]. The polymorphic band amplified in CMS carpelloid stamen materials was 420 bp, which suggests that the original CMS source came from OguCMSHY, OguCMSR_1-2_, or NigCMS and was transferred to broccoli. Alignment analysis of the sequences showed that the deleted sequences in broccoli were not at the same location as in cabbage. This will benefit the selection of CMS types and backcross breeding in broccoli; moreover, it will also provide a basis for evolutionary studies in crucifers.

Furthermore, the sequences of the products were located in *orf108* or *orf125* coding regions of mitochondrial genomes in many *Brassica* species. Compared with the maintainers, the carpelloid stamen materials showed a deletion of 51 nucleotides, suggesting that the deletion of mitochondrial nucleotides could explain the carpelloid stamen phenotype. Thus, it is likely that *orf108* or *orf125* encodes genes involved in the development of floral organs. Our findings provide an important reference for further study on the molecular mechanism of the interaction between the nucleus and cytoplasm in floral organ development.

In conclusion, we have identified a useful molecular marker to detect the cytoplasm of carpelloid stamens in broccoli. The results of our study are valuable for improving the efficiency of broccoli breeding and also for forming a solid basis for further study of the molecular mechanisms underlying the CMS carpelloid stamen phenomenon.
